# TKI-Resistant Renal Cancer Secretes Low-Level Exosomal miR-549a to Induce Vascular Permeability and Angiogenesis to Promote Tumor Metastasis

**DOI:** 10.3389/fcell.2021.689947

**Published:** 2021-06-10

**Authors:** Zuodong Xuan, Chen Chen, Wenbin Tang, Shaopei Ye, Jianzhong Zheng, Yue Zhao, Zhiyuan Shi, Lei Zhang, Huimin Sun, Chen Shao

**Affiliations:** ^1^Medical College, Xiamen University, Xiamen, China; ^2^School of Public Health, Xiamen University, Xiamen, China; ^3^Department of Urology Surgery, Xiang’an Hospital, Xiamen University, Xiamen, China

**Keywords:** TKI-resistant, clear cell renal cell carcinoma, exosome, microRNA, HIF1α, vascular endothelial permeability, metastasis

## Abstract

Tyrosine kinase inhibitors (TKI)-resistant renal cancer is highly susceptible to metastasis, and enhanced vascular permeability promotes the process of metastasis. To evaluate the effect of cancer-derived exosomes on vascular endothelial cells and clarify the mechanism of metastasis in TKI-resistant renal cancer, we studied the crosstalk between clear cell renal cell carcinoma (ccRCC) cells and human umbilical vein endothelial cells (HUVECs). Exosomes from ccRCC cells enhanced the expression of vascular permeability-related proteins. Compared with sensitive strains, exosomes from resistant strains significantly enhanced vascular endothelial permeability, induced tumor angiogenesis and enhanced tumor lung metastasis in nude mice. The expression of miR-549a is lower in TKI-resistant cells and exosomes, which enhanced the expression of HIF1α in endothelial cells. In addition, TKI-resistant RCC cells reduced nuclear output of pre-miR-549a via the VEGFR2-ERK-XPO5 pathway, and reduced enrichment of mature miR-549a in cytoplasm, which in turn promoted HIF1α expression in RCC, leading to increased VEGF secretion and further activated VEGFR2 to form a feedback effect. miR-549a played an important role in the metastasis of renal cancer and might serve as a blood biomarker for ccRCC metastasis and even had the potential of becoming a new drug to inhibit TKI-resistance.

## Introduction

Tyrosine kinase inhibitors (TKI) is the main treatment for advanced renal cancer, but most patients will eventually develop TKI-resistant RCC then metastasis occurs after 6–15 months, in which hematogenous metastasis is the main route (Wyler et al., 2014). Sorafenib is the first multi-targeted TKI drug for the treatment of metastatic renal cell carcinoma (mRCC) to inhibit Raf/MEK/ERK signaling pathway and VEGFR to achieve multiple anti-tumor effects (Wilhelm et al., 2004). In recent years, sunitinib has been recommended as first-line treatment for ccRCC and systemic treatment for non-clear cell RCC according to National Comprehensive Cancer Network (NCCN) guidelines. However, sunitinib is more toxic than sorafenib. In Asian patients, sorafenib may be more appropriate than sunitinib (Deng et al., 2019). Therefore, we conducted a study on sorafenib-resistant clear cell renal cancer. Tumor metastasis is a complex multistep process. Tumor cells detach from the primary foci, migrate and invade the extracellular matrix, enter the blood vessel, survive in the blood, exude and disseminate in the target organ, grow and form metastatic foci (Reymond et al., 2013; Paul et al., 2017). The increase of vascular permeability is tightly associated to the tumor cells entering the blood circulation and then colonizing at the distal organ to form metastatic foci (García-Román and Zentella-Dehesa, 2013; Harrell et al., 2014). Exosomes are a subset of extracellular vesicles (EVs) with an average diameter of about 100 nm, and their components include nucleic acids, proteins, lipids, amino acids, and metabolites (Xu et al., 2018; Kalluri and LeBleu, 2020). After being released from tumor cells, exosomes are ingested by adjacent or distal cells, then the contained miRNAs regulate tumor immunity and microenvironment (Sun et al., 2018; Meng et al., 2019). Studies have found that exosomal miRNAs from breast, liver and colon cancer affect endothelial cells to promote metastasis (Zhou et al., 2014; Fang et al., 2018; Zeng et al., 2018). However, the mechanism by which TKI-resistant renal cancer cells induce vascular endothelial cell permeability changes to promote metastasis remains unclear. Here, we found that TKI-resistant cells of renal clear cell carcinoma had lower expression level of miR-549a than sensitive cells. miR-549a was delivered to vascular endothelial cells via exosomes to inhibit the expression of HIF1α. Therefore, compared with sensitive strain, resistant strain with lower levels of miR-549a had weaker effects on HIF1α, which enhanced permeability of vascular endothelium, and promoted angiogenesis, which in turn promoted tumor metastasis. In addition, in exploring the upstream regulatory mechanism of miR-549a in TKI-resistant renal cancer cells, we found that there was a positive feedback in renal cancer cells. Activation of the VEGFR2-ERK-XPO5 pathway inhibited the nuclear export of pre-miR-549a, and reduction of mature miR-549a in the cytoplasm promoted HIF1α expression to enhance VEGF secretion and then activated VEGFR2. The above results demonstrated the key role of miR-549a in promoting vascular permeability and angiogenesis in TKI-resistant renal cancer, and provided a new idea for the treatment.

## Materials and Methods

### Cell Lines and Cell Culture

Human umbilical vein endothelial cells (HUVEC), renal clear cell carcinoma cells (786-O) and human renal epithelial cells (293T) were purchased from the American Type Culture Collection (ATCC). 786-O-SR was induced from 786-O with sorafenib (Solarbio, China). HUVECs were cultured in the ECM medium (ScienCell, United States) supplemented with 15% fetal bovine serum (ScienCell, United States). 786-O and 786-O-SR cell lines were cultured in 1640 medium (GIBCO, United States) supplemented with 10% fetal bovine serum (GIBCO, United States), and 15 μm sorafenib was added to 786-O-SR culture to maintain drug resistance. 293T cells were cultured in DMEM medium (GIBCO, United States) containing 10% fetal bovine serum. The cells were cultured in humidified air at 37°C and 5%CO_2_ with 100 U/ml penicillin and 100 μg/ml streptomycin (GIBCO, United States). For hypoxia culture, cells were placed in a hypoxic incubator at 37°C in a humidified 1% O2, 5% CO2 environment, with the balance provided by N2 for 24 h. FBS, used for CM collection, exosome separation and endothelial cell treatment, was centrifuged overnight at 100,000 *g* at 4°C.

### Preparation of Conditioned Medium (CM)

Conditioned medium of renal cell carcinoma cells cultured in ECM were collected and stored at –80°C. When incubated with HUVEC, the collected CM was supplemented with 10% exosome-depleted FBS.

### Isolation, Characterization, and Quantification of Exosomes

Exosomes were isolated from CM of renal carcinoma cells, which were cultured in 1640 supplemented with 10% exosome-depleted FBS, analyzed by transmission electron microscopy (TECNAI spirit, Fei, Netherlands) and particle size analyzer (Nicomp 380 N3000, PSS). The number of cells was counted to appropriately correct the CM volume used to separate the exosomes. The protein concentration of the harvested exosomes was detected by BCA Protein Quantification Kit (Yeasen, China). For cell treatment, exosomes collected from 5 × 10^6^ cells (equivalent to 2 μg exosomes) were added to 2 × 10^5^ endothelial cells.

### Cellular Internalization of Exosomes

The exosomes were labeled with BODIPY TR ceramide (Thermo Fisher Scientific, United States) and then resuspended in 10% exosome-depleted FBS-ECM, added to HUVECs at 80% confluence, incubated for 4 h, and imaged under fluorescence microscope.

### Real Time PCR

Total RNA was extracted from the cells using TRIzol RNA Isolation Reagents (ThermoFisher, United States). All-in-One^TM^ miRNA First-Strand cDNA Synthesis Kit (GeneCopoeia, United States) was used for miRNA reverse transcription. PrimeScript^TM^ RT Master Mix (Clonech Laboratories, United States) was used for universal gene. Real time PCR was performed by SYBR Green PCR Master Mix (Applied Takara, Japan) and CFX96 deep hole real-time PCR detection system (BioRad, United States). The sequences of all primers are listed in Supplementary Figure 1F.

### Gel Electrophoresis

RT-PCR samples were electrophoretic on 1% agarose gel and photographed under ultraviolet light using BioRad imager. BioRad quantity one imaging software was used to quantify the strip strength.

### Protein Extraction and Western Blotting

Cells and exosomes were prepared in RIPA buffer (KeyGEN BioTECH, China) and quantified by BCA Protein Quantification Kit (Yeasen, China). Nuclear protein was extracted by CelLytic^TM^ NuCLEAR^TM^ Extraction Kit (Sigma-Aldrich, United States). Then, the lysate was transferred to the PVDF membrane (Millipore, United States) by SDS-PAGE. Then the membrane were incubated with primary antibodies at 4°C overnight. The following antibodies were used: vimentin (Cell Signaling, 5741t, 1:1000 dilution), β-catenin (Cell Signaling, 8480t, 1:1000 dilution), *E*-cadherin (Cell Signaling, 3195t, 1:1000 dilution), *N*-cadherin (Cell Signaling, 13116t, 1:1000 dilution), claudin1 (Cell Signaling, 13255t, 1:1000 dilution), ZO-1 (Cell Signaling, 8193t, 1:1000 dilution), β-actin (Cell Signaling, 4970s, 1:1000 dilution), CD81 (Cell Signaling, 10037s, 1:1000 dilution), TSG101 (Invitrogen, ma1-23296, 1:2000 dilution), HIF1α (Cell Signaling, 36169s, 1:1000 dilution), Xpo5 (Cell Signaling, 12565s, 1:1000 dilution), VEGFR2 (Cell Signaling, 9698s, 1:1000 dilution), ERK 1/2 (Cell Signaling, 4696s, 1:2000 dilution), Hillstone H3 (Cell Signaling, 14269s, 1:1000 dilution). After incubation with HRP linked antibody (Cell Signaling, 7076s or 7074s, 1:3000 dilution), the chemiluminescence signal was detected using the hypersensitive ECL Western blotting detection reagent (Seville Biology).

### RNA Oligoribonucleotides and Vectors

miR-549a-3p/miR-549a-5p mimics and miR-549a-3p/miR-549a-5p inhibitors and their negative controls (NC and anti-NC, respectively) were provided by GenePharma, China. The sequences of the above miRNA mimics and inhibitors are listed in Supplementary Figure 1H. GFP plasmid and pcDNA plasmid were purchased from Sino Biological, China. The sea cucumber firefly dual luciferase reporter system (GenePharma, China) was used to verify whether the 3′-UTR of HIF1α mRNA was targeted by miR-549a.

### Cell Transfection

Lipofectamine 3000 (ThermoFisher, United States) was used for the transfection of miR-549a-3p/miR-549a-5p mimics, miR-549a-3p/miR-549a-5p inhibitors and their negative controls (NC and anti-NC, respectively) and co-transfection of RNA duplexes with plasmid DNA.

### Transendothelial Invasion Assay, Migration Assay and Angiogenesis Assay

For transendothelial invasion assays, *in vitro* endothelial permeability was assessed by counting the amount of 786-O-GFP that passed through a single layer of HUVECs with or without exosome treatment. For migration assays, exosome-treated HUVECs were suspended in serum-free medium and seeded into transwell chamber with 8-micron pore size (BD Biosciences, United States). The medium containing 15% FBS was placed in the bottom chamber. After 12 h, cells that migrated through the membrane and adhered to the submucosal surface were stained with hematoxylin and counted under light microscopy in four random fields of view (200x). For tube formation assays, the Matrigel matrix (Corning, United States) was laid in a 24-well plate and incubated at 37°C for 30 min to polymerize the matrix. The treated HUVECs were seeded on the matrix gel-coated holes. The plates were then incubated at 37°C in a 5%CO_2_ humidified atmosphere. Tube formation was observed with a microscope after 12 h. The tube forming ability is determined by measuring the number of tubes. Each experiment was repeated three times.

### Luciferase Activity Assay

The HIF1α 3′UTR plasmid was co-transfected into cells with either miR-549a-3p or miR-549a-3p mimics. Luciferase activity was measured by Dual-Luciferase Reporter Assay System (Promega, United States) 48 h after transfection. All assays were performed in triplicate and each experiment was repeated three times.

### Animal Models

Six-week-old male athymic BALB/c nude mice were purchased from the Laboratory Animal Center of Xiamen University (Xiamen, China) and raised in a pathogen-free environment. All protocols for animal research were reviewed and approved by the Laboratory Animal Center of Xiamen University (Ethics No. XMULAC20200039). For tumor metastasis assay, 2 × 10^6^ 786-O cells were injected into nude mice via tail vein. Six-week-old nude mice was injected into the tail vein with 5 μg exosomes every other day for 2 weeks. The control group was injected with equal volume PBS. After 15 and 30 days, respectively, the mice were sacrificed and the lungs were removed for examination.

### Immunohistochemistry

Paraffin-embedded tissue blocks were cut into 2.5-μm sections and transferred to slides. Sections were immersed in 3% hydrogen peroxide to block endogenous peroxidase activity and incubated with primary antibody overnight at 4°C. Subsequently, horseradish peroxidase-conjugated secondary antibodies (DakoCytomation, Glostrup, Denmark) were applied and incubated at room temperature for 1 h. CD34 expression was visualized by using DAB and counterstained with hematoxylin. The following primary antibodies were used: CD34 (Abcam, ab81289, 1:200 dilution).

### Immunofluorescence

Cells grown on glass coverslips were fixed with 4% paraformaldehyde for 10 min at room temperature. The cells were rinsed twice with PBS. Blocking buffer (DakoCytomation, Glostrup, Denmark) was added for 30 min, and then stained with primary antibodies and fluorescent second antibody. The following antibodies were used: VEGFR2 (Cell Signaling, 9698S, 1:800 dilution), ERK 1/2 (Cell Signaling, 4696S, 1:100 dilution). Anti-mouse IgG (Alexa Fluor #594 Conjugate) (Cell Signaling, 8890, 1:2000 dilution), Anti-rabbit IgG (Alexa Fluor #488 Conjugate) (Cell Signaling, 4412, 1:2000 dilution).

### Statistical Analysis

Statistical analysis was performed using GraphPad Prism 7.0 software. Quantitative values of all experiments were expressed as mean standard deviation. Differences between sample groups were analyzed by one-way ANOVA or independent sample *T*-test. *P* < 0.05 was considered statistically significant. Adobe Illstrator CC, Adobe Photoshop CC and Image J software were used for the figure.

## Results

### Renal Clear Cell Carcinoma TKI Resistant Cell Strain 786-O-SR Is Resistant to Sorafenib

**FIGURE 1:**
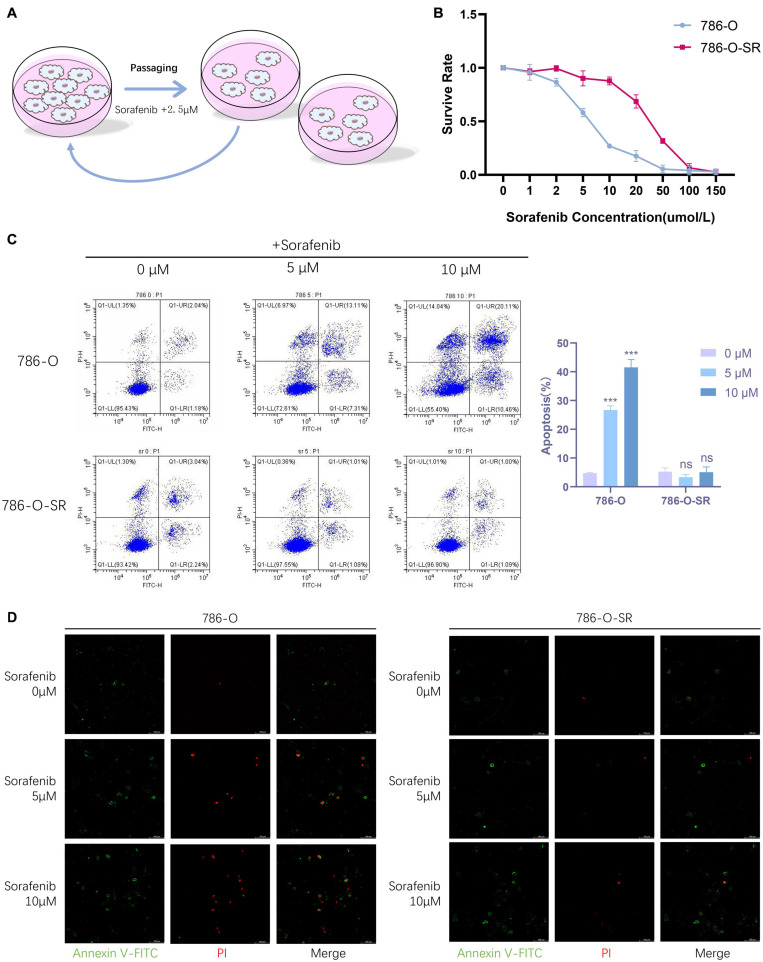
Renal clear cell carcinoma TKI resistant cell strain 786-O-SR is resistant to sorafenib. **(A)** Induction process of TKI-resistant cell line 786-O-SR. **(B)** CCK8 assay of cell viability of 786-O and 786-O-SR at different sorafenib concentrations. **(C)** Flow cytometry assay of apoptosis of 786-O and 786-O-SR in response to different sorafenib concentrations (detected by annexin-V combined with PI). **(D)** Apoptosis situations of 786-O and 786-O-SR at different sorafenib concentrations observed by fluorescence microscopy. Mean ± SEM are provided (*n* = 3). ***P < 0.001; ns, not significant according to two-tailed Student’s *t*-test.

To derive sorafenib resistant cell strain, we continuously cultured renal clear cell carcinoma cells (786-O), which is sensitive to sorafenib, in a stepwise manner by increasing the concentration of sorafenib, the concentration of sorafenib started at 5 μM and increased by 2.5 μM per generation to finally reach a concentration of 15 μM, from which a sorafenib resistant renal clear cell carcinoma cell line (786-O-SR) was induced (Figure 1A). 786-O-SR was cultured at 15 μM sorafenib to maintain its drug resistance.

To identify the TKI resistant renal clear cell carcinoma cell strain (786-O-SR) derived from 786-O induced by sorafenib, we assessed cell viability of 786-O versus 786-O-SR at different sorafenib concentrations by CCK8 assay, and the results showed that the cell viability of 786-O-SR did not alter significantly at low concentrations of sorafenib and decreased when sorafenib concentrations exceeded 10 μM, whereas the TKI sensitive strain 786-0 exhibited a dramatic inhibition of cell viability under the treatment of low concentrations of sorafenib (Figure 1B). Furthermore, we examined the apoptosis of 786-O and 786-O-SR at different sorafenib concentrations using annexin-V in combination with PI, and the results showed that 786-O exhibited a higher apoptosis rate with sorafenib treatment and positively correlated with sorafenib concentration, while the apoptosis of 786-O-SR was unaffected under sorafenib treatment at a range of concentrations (Figure 1C). Under fluorescence microscopy, an increasing trend of 786-O early apoptotic cells (annexin-V single positive), late apoptotic cells (annexin-V and PI double positive) as well as necrotic cells (PI single positive) could be observed with increasing sorafenib concentration, while the change of 786-O-SR counterpart was less obvious (Figure 1D).

### Exosomes Derived From Clear Cell Renal Cell Carcinoma Cells Increase the Permeability of the Endothelial Cells

**FIGURE 2:**
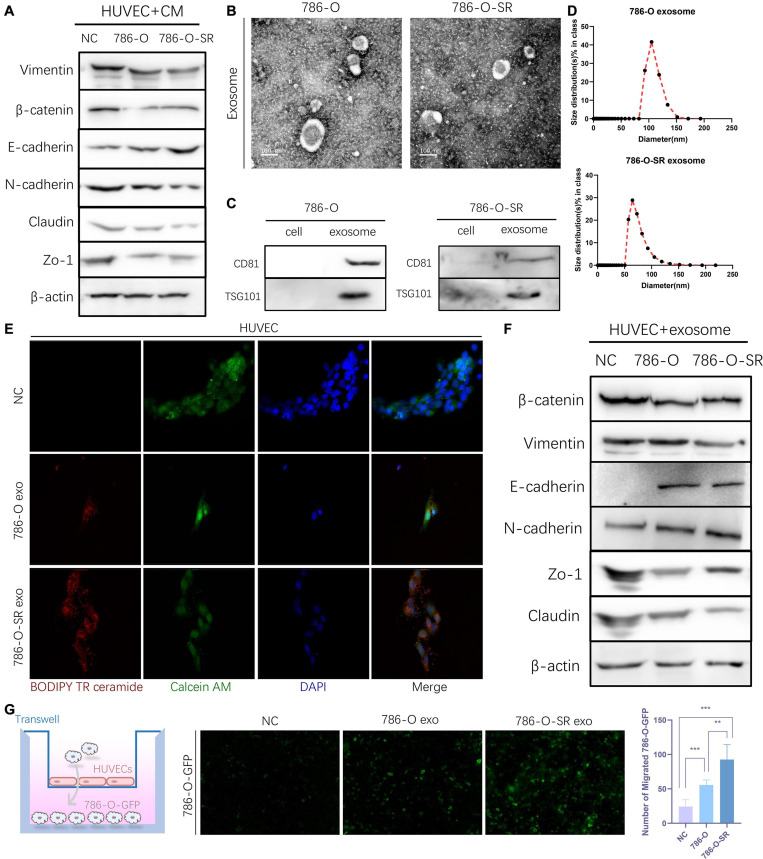
Exosomes derived from Clear cell renal cell carcinoma cells increase the permeability of the endothelial cells. **(A)** Western blot analysis of Vimentin, β-catenin, *E*-cadherin, *N*-cadherin, Zo-1, Claudin expression in HUVECs incubated with CM of 786-O and 786-O-SR. **(B)** Transmission electron microscopy of exosomes derived from 786-O and 786-O-SR. Scale bar, 100 nm. **(C)** Western blotting analysis of CD81 and TSG101 in 786-O, 786-O-SR and their exosomes. **(D)** Nanoparticle tracking analysis of the size distribution and median diameter of particles per μg exosomes from 786-O and 786-O-SR. **(E)** The presence of BODIPY TR ceramide fluorescence in HUVECs after adding dye-labeled exosomes derived from 786-O and 786-O-SR cells for 48 h. HUVECs incubated with PBS were used as a negative control. Red: BODIPY TR ceramide; Green: Calcein AM; Blue: DAPI. **(F)** Western blot analysis of β-catenin, Vimentin, *E*-cadherin, *N*-cadherin, Zo-1, Claudin expression in HUVECs incubated with exosomes of 786-O and 786-O-SR. **(G)** Transendothelial invasion assay analysis of the number of GFP-expressing 786-O cells that invaded through HUVECs monolayers cultured with exosome derived from 786-O or 786-O-SR. Mean ± SEM are provided (*n* = 3). **P < 0.01, ***P < 0.001, according to two-tailed Student’s *t*-test. exo, exosomes; NC, negative control.

To understand the effect ccRCC exert on endothelial cells and whether sorafenib-sensitive (786-O) and TKI-resistant (786-O-SR) cells have differential effects, HUVECs were cultured with CM of 786-O or 786-O-SR. After CM treatment, HUVECs showed decreased expression of β-catenin, Vimentin, ZO-1 and Claudin and up-regulated expression of *E*-cadherin, and the change was more significant with treatment of CM from 786-O-SR than 786-O (Figure 2A). Vimentin is a type III intermediate filament protein which plays a role in stabilizing and enhancing endothelial matrix adhesion (Tsuruta and Jones, 2003). β-catenin inhibits VE-cadherin hydrolysis (Komarova and Malik, 2010), promotes the formation and maintenance of adherent junctions. ZO-1 and Claudin are tight junction proteins. *N*-cadherin inhibits vascular protective repair in epithelial cells (Jian et al., 2016). The above changes indicated that the permeability of HUVECs was enhanced after CM treatment, and the effect of 786-O-SR was more obvious.

Exosome is an important tool for intercellular communication with diameters from tens to hundreds of nanometers. We extracted and identified the exosomes of 786-O and 786-O-SR. Vesicle-like structures (Figure 2B) were observed under the electron microscopy, and the expression of CD81 and TSG101 (Figure 2C) was detected by WB. The particle size of 786-O exosomes was slightly larger than that of 786-O-SR, but all were within the diameter range of exosomes (Figure 2D). After co-incubation with exosomes, the changes of *β*-catenin, Vimentin, ZO-1, Claudin and *N*-cadherin in HUVECs were the same as those after CM treatment (Figure 2F). Transendothelial invasion assay showed that the number of 786-O-GFP crossing monolayer HUVECs increased after exosome treatment, and the effect of 786-O-SR exosome was more significant (Figure 2G). To confirm the absorption of exosomes derived from 786-O/786-O-SR by HUVECs, HUVECs were incubated with exosomes labeled with BODIPY TR ceramide, and red fluorescence signal was transferred to HUVEC (Figure 2E), but not to control group. Thus, ccRCC exosomes have an impact on vascular endothelial cell permeability, and TKI-resistant renal cancer has a greater impact on vascular permeability.

### Exosomal miR-549a Affects Vascular Permeability

**FIGURE 3:**
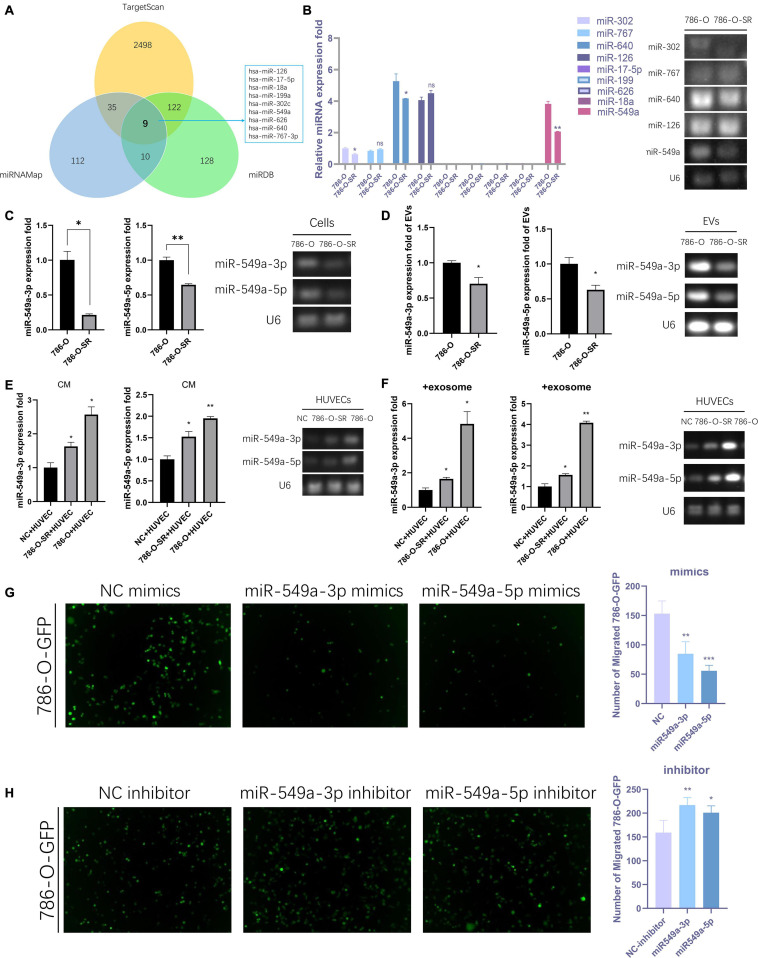
Exosomal miR-549a affects vascular permeability. **(A)** The Wayne figures of overlapping and different miRNAs which target HIF1α according to TargetScan, miRNAMap and miRDB. **(B)** RT-PCR analysis of 9 miRNAs (which target HIF1α) expression in 786-O/786-O-SR cells and gel electrophoresis of PCR products. **(C–F)** RT-PCR and gel electrophoresis of PCR products analysis of miR-549a-3p/miR-549a-5p expression in 786-O/786-O-SR cells **(C)**, exosomes **(D)**, HUVECs treated with 786-O/786-O-SR CM **(E)** or exosomes **(F)**. **(G,H)** Transendothelial invasion assay analysis of the number of GFP-expressing 786-O cells that invaded through HUVECs monolayers transfected with miR-549a-3p/miR-549a-5p mimics **(G)** or miR-549a-3p/miR-549a-5p inhibitor **(H)**. Mean ± SEM are provided (*n* = 3). *P < 0.05, **P < 0.01, ***P < 0.001, according to two-tailed Student’s *t*-test. ns, not significant.

We sequenced the VEGF pathway of HUVEC cells treated with exosomes from 786-O and 786-O-SR cells and found that there were a series of differentially expressed proteins (Supplementary Figure 1A). 17 proteins were over-expressed in 786-O-SR treatment group and four proteins were under-expressed (Supplementary Figure 1B), among which HIF1α expression level was the most significant after excluding the influence of oxygen conditions during cultivation process (Supplementary Figure 1B). MiRNAs are a class of single-stranded small RNAs about 22 NT long, processed from hairpin structural transcripts produced endogenously in cells (Kim, 2005). The main function of miRNAs is to inhibit the expression of downstream genes then weaken or eliminate their function. Moreover, miRNAs achieve intercellular communication through exosomes. Nine upstream miRNAs of HIF1α were preliminarily screened by combining the predicted results of Targetscan, miRDB, and miRNAMap databases (Figure 3A). Further examination of the expression of these miRNAs in 786-O and 786-O-SR revealed that miR-17-5p, miR-199, miR-626, and miR-18a were not expressed in ccRCC. No significant difference was observed in the expression of miR-767 and miR-126 between sensitive and resistant strains. miR-302, miR-640, and miR-549a showed differential expression, among which the difference of miR-549a was the most significant (Figure 3B).

The expression level of miR-549a-3p/miR-549a-5p of 786-O was higher than 786-O-SR (Figure 3C), and same trend difference was observed in its exosomes (Figure 3D). Treated with CM or exosomes of 786-O-SR/786-O, the level of miR-549a-3p/miR-549a-5p of HUVEC increased, and 786-O had a greater effect (Figures 3E,F).

To verify whether miR-549a affects HUVEC permeability, a transendothelial invasion assay was performed. Transfected with NC, mimics or inhibitor, the number of 786-O-GFP was significantly different. miR-549a mimics resulted in a reduction, and the effect of miR-549a-5p mimics was more obvious (Figure 3G). Opposite result was obtained in miR-549a inhibitor treated group (Figure 3H). This suggested that miR-549a-3p and miR-549a-5p inhibited the permeability of HUVEC, which explained why TKI-resistant renal cancer cells (786-O-SR) with low expression of miR-549a exerted stronger permeability-promoting effect on HUVECs, whereas sensitive strains (786-O) with higher expression of miR-549a had weaker effect.

However, the permeability of HUVECs treated with CM or exosomes of renal cancer cells was enhanced compared with that of the control group (i.e., HUVEC without exogenous input of miR-549a) (Figures 2A,F,G), suggesting that tumor-derived exosomes had some factors that positively regulated vascular permeability. HUVEC naturally expressed low level of *E*-cadherin, a key molecule in cell-cell adhesions (van Roy and Berx, 2008), which increased after treatment with renal cancer exosomes (Figure 2F). It was reported that *E*-cadherin localized on the surface of exosome membrane was transported to endothelial cells to promote angiogenesis (Tang et al., 2018). *E*-cadherin was expressed both in 786-O/786-O-SR cells and their exosomes, and 786-O-SR expression was higher (Supplementary Figure 1C). This suggested that renal cancer exosomes transmitted *E*-cadherin to endothelial cells. Studies have suggested that *E*-cadherin regulated HIF1α (Maroni et al., 2015; Liang et al., 2016), which may be one of the mechanisms by which renal cancer exosomes promote vascular permeability.

### Exosomal miR-549a Affects Angiogenesis and Endothelial Cell Migration

**FIGURE 4:**
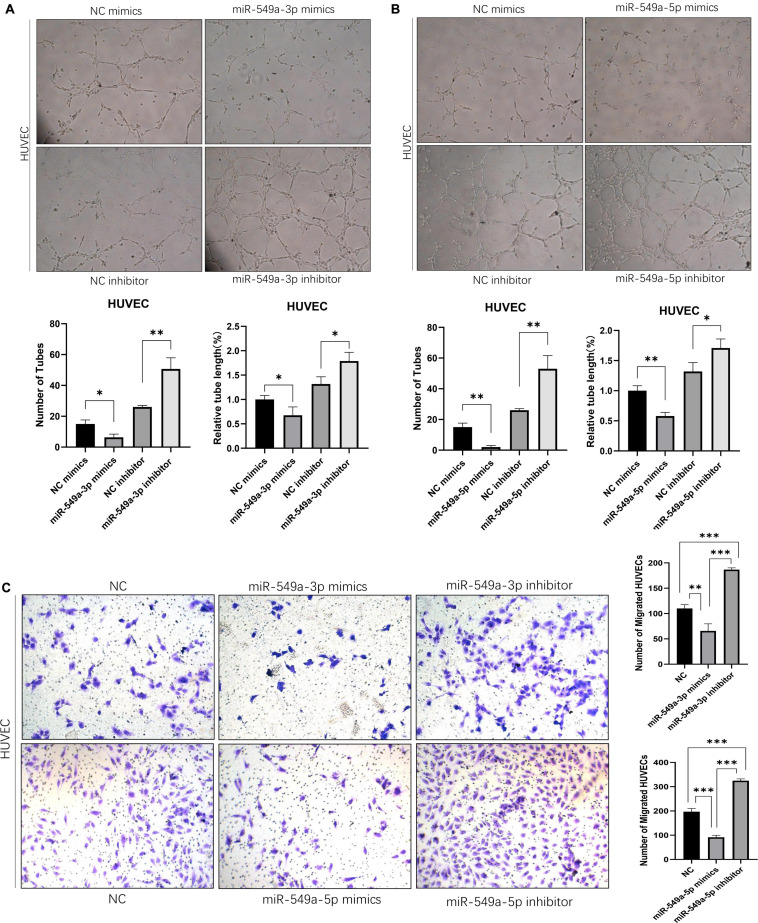
Exosomal miR-549a affects angiogenesis and endothelial cell migration. **(A,B)** Effect of miR-549a-3p mimics/inhibitor **(A)**, miR-549a-5p mimics/inhibitor **(B)** on tube formation ability of HUVECs by tube formation assay. Mean ± SEM are provided (*n* = 3). **(C)** Effect of miR-549a-3p/miR-549a-5p mimics and inhibitor treatment on migration of HUVECs. Mean ± SEM are provided (*n* = 3). ^∗^*P* < 0.05, ^∗∗^*P* < 0.01, ^∗∗∗^*P* < 0.001 according to two-tailed Student’s *t*-test.

Angiogenesis plays a key role in tumor progression. In the primary lesion, angiogenesis ensures the nutrient supply of tumor cells. Formation of secondary metastatic foci by tumor cells requires the formation of pre-metastatic niche, in which angiogenesis is the key step. In addition, neovascularization is characterized by high vascular permeability, so the enhancement of angiogenesis also leads to the increase of overall vascular permeability. To test the effect of miR-549 expression upon angiogenesis, we performed a tube-formation assay of HUVEC *in vitro*. The results showed that miR-549a-3p mimics significantly reduced the lumen structure formed by HUVEC, while miR-549a-3p inhibitor enhanced the tube-forming ability of HUVEC (Figure 4A). Similarly, miR-549a-5p mimics led to a decrease in the tubulogenic capacity of HUVEC, whereas inhibitor did the opposite (Figure 4B).

Since endothelial cell migration is also essential for angiogenesis and leads to increased vascular leakage, we evaluated the effect of miR-549a on HUVEC migration. After treating HUVECs with mimics and inhibitors of miR-549a-3p, miR-549a-5p, we found that, miR-549a-3p and miR-549a-5p significantly attenuated the migration ability of HUVECs, while the migration ability of HUVECs was improved after inhibiting miR-549a-3p and miR-549a-5p (Figure 4C). These results indicate that miR-549a weakens angiogenesis and endothelial cell migration.

### Renal Cancer Exosomes Promote ccRCC Metastasis

**FIGURE 5:**
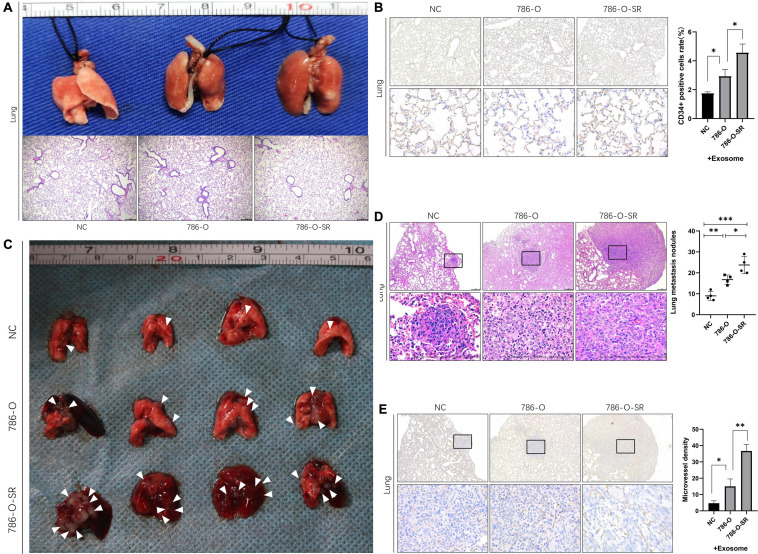
Renal cancer exosomes promote ccRCC metastasis. **(A)** The mice were injected with 786-O cells via tail vein after exposure to 786-O or 786-O-SR exosomes. The lungs were removed for H&E staining after 15 days. **(B)** IHC staining for CD34-positive cells. Mean ± SEM are provided (*n* = 3). **(C)** The lungs were removed after 30 days. Lung metastatic sites were indicated by arrows. **(D)** The number of lung metastatic sites was counted under the microscope. Mean ± SEM are provided (*n* = 4). Scale bar 200 μm. **(E)** Effect of exosomes of 786-O/786-O-SR on introtumoral microvessel density. The number of microvessel was counted under the microscope. Mean ± SEM are provided (*n* = 3). ^∗^*P* < 0.05, ^∗∗^*P* < 0.01, ^∗∗∗^*P* < 0.001 according to two-tailed Student’s *t*-test.

To determine whether exosomes promote renal cancer cell metastasis *in vivo*, we injected ccRCC cells into mice via the tail vein, daily treated with 786-O/786-O-SR exosomes, and monitored tumor metastasis. Mice were sacrificed on day 15, and the lungs were removed and subjected to histological examination (Figure 5A). HE showed metastasis sites were not formed in lung (Figure 5A), but the microvessel density (MVD) of the lung tissue had changed. Both 786-O/786-O-SR exosomes lead to an increase of CD34-positive cell rate, of which 786-O-SR exosomes had a greater impact (Figure 5B). This suggests that before tumor metastases, exosomes modify the microenvironment of distal organs, inducing enhanced angiogenesis to form a pre-metastatic niche which is conducive to tumor colonization.

After the occurrence of metastasis, the lungs of mice were removed on day 30. The representative gross morphology of lung metastasis was displayed. More metastasis foci were observed after exosome treatment, and the effect of 786-O-SR exosome was more significant (Figure 5C). Moreover, the metastases of 786-O-SR exosome-treated tumors had invaded the mediastinum and pleura. HE showed that the exosome-treated group had more metastases (Figure 5D). Tumor MVD was higher in the exosome-treated group than in the control group, with 786-O-SR group having the highest MVD (Figure 5E).

### miR-549a Silences HIF1α in HUVECs

**FIGURE 6:**
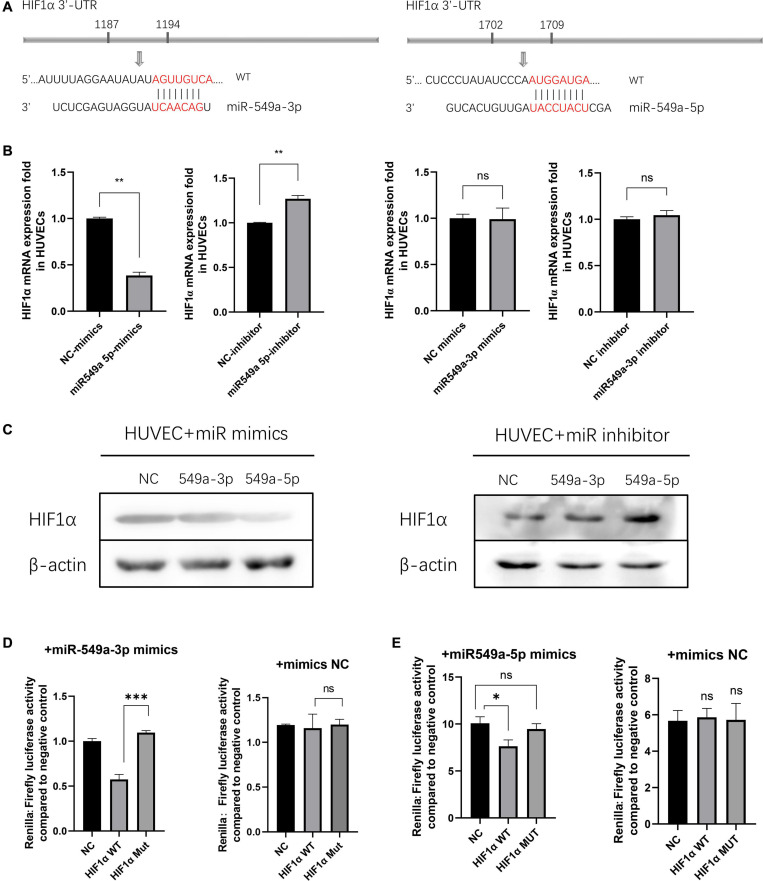
miR-549a silences HIF1α in HUVECs. **(A)** The miR-549a-3p and miR-549a-5p binding sites in the 3′-UTR of HIF1α were predicted. **(B)** RT-PCR analysis of HIF1α RNA expression in HUVECs treated with miR-549a-3p/miR-549a-5p mimics or inhibitor. **(C)** Western blot analysis of HIF1α expression in HUVECs treated with miR-549a-3p/miR-549a-5p mimics or inhibitor. **(D,E)** Dual-luciferase reporter gene assay showed that miR-549a-3p/miR-549a-5p inhibited the luciferase activity of reporter containing wild-type but not mutant 3′-UTR of HIF1α. Mean ± SEM are provided (*n* = 3). *P < 0.05, **P < 0.01, ***P < 0.001; ns, not significant according to two-tailed Student’s *t*-test.

To validate the regulatory effect of miR-549a on HIF1α, we compared their sequences by BLAST. A base match was found between HIF1α mRNA 3′-UTR and miR-549a-3p as well as miR-549a-5p, respectively (Figure 6A).

There are three main effects of miRNA: transcriptional repression and cleavage or degradation of mRNA, while the miRNA *in vivo* generally does not match the target gene well, so this regulation is not common in animals (Mohr and Mott, 2015). The possibility of miRNA-mediated mRNA degradation is higher. When silenced, the decapping and tail removal reaction of the mRNA are triggered, then cause the degradation (Jonas and Izaurralde, 2015). RT-PCR showed that miR-549a-5p reduced the HIF1α mRNA level in HUVECs, while miR-549a-3p had not effect (Figure 6B). Mimics of miR-549a-3p/miR-549a-5p inhibited HIF1α protein level, while their inhibitor effect was opposite (Figure 6C). The above results indicated that both miR-549a-3p and miR-549a-5p reduced HIF1α protein level. MiR-549a-3p inhibits the translation process of HIF1α mRNA but does not decrease its mRNA level, while miR-549a-5p induces the degradation of HIF1α mRNA.

To verify the binding effect of miR-549a to HIF1α mRNA 3′-UTR, a dual-luciferase reporter gene assay was performed. The luciferase activity of 3′ UTR of HIF1α was suppressed notably by miR-549a-3p, while mutant HIF1α had no such effect (Figure 6D). Similarly, miR-549a-5p had also been proved to bind to the 3’-UTR region of HIF1α mRNA (Figure 6E).

Since HIF1α is regulated by environmental oxygen levels, in order to verify the regulatory effect of miR-549a on HIF1α under hypoxia, we detected HIF1α levels in HUVECs cultured under hypoxia. The results showed that the changes in HIF1α RNA and protein levels were consistent with those in normoxia (Supplementary Figure 1E). Moreover, HIF1α was not detected in exosomes derived from renal cancer, excluding the possibility that renal cancer derived exosomes carrying HIF1α protein to recipient endothelial cells to achieve regulation (Supplementary Figure 1I).

The above results show that miR-549a binds to the 3′-UTR region of HIF1α mRNA to inhibit its translation process, in which miR-549a-5p leads to a decrease in HIF1α mRNA levels while miR-549a-3p does not, but ultimately both lead to a significant reduction in HIF1α protein levels in target cells, and the process is universal under different oxygen conditions.

### Erk2 Regulates the Output of miR-549a via XPO5

Exportin-5 (XPO5) is a miRNA transport protein present in the nucleus. In the nucleus, primary microRNAs are sheared by nuclease Drosha to form pre-miRNAs with stem-loop structure of about 70 nucleotides. XPO5 transports the pre-miRNAs from the nucleus to the cytoplasm. Sheared by the nuclease, Dicer, in the cytoplasm, pre-miRNAs become mature miRNAs with about 20–25 nucleotides and get bioactivity (Clancy et al., 2019). Given the critical role of nuclear export of pre-miRNAs in the biological functions of miRNAs, any changes affecting XPO5 affect the expression of miRNAs, thus having a profound impact on tumorigenesis and progression (Wu et al., 2018). Erk (mainly Erk2) decreases the binding ability of p-XPO-5 to pre-miRNA by phosphorylating XPO5 at T345/S416/S497, resulting in a decrease in the extranuclear export of pre-miRNA (Sun et al., 2016).

**FIGURE 7:**
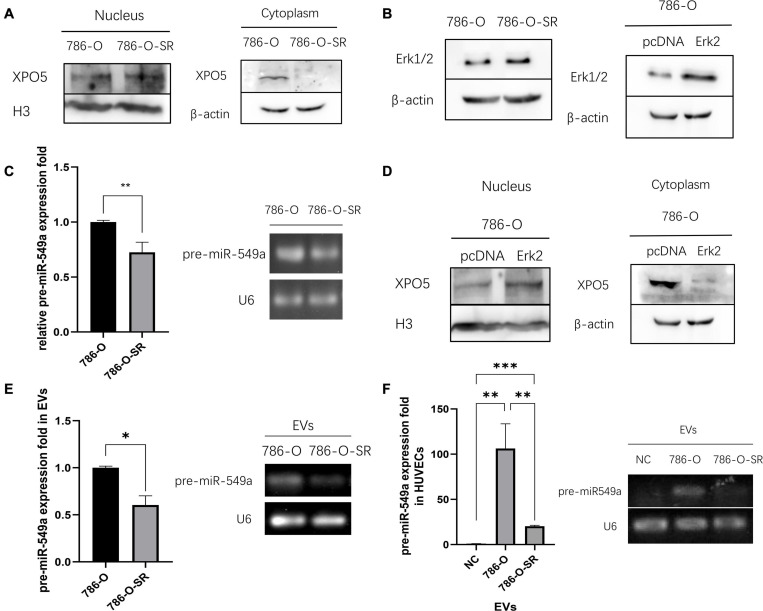
Erk2 regulates the output of miR-549a via XPO5. **(A)** Cytosolic and nuclear protein were prepared and analyzed for XPO5 expression in 786-O and 786-O-SR by Western blot. **(B)** Western blot analysis of Erk1/2 expression in 786-O and 786-O-SR and the detection of overexpression efficiency of Erk2 in 786-O. **(C)** RT-PCR and gel electrophoresis of PCR products analysis of pre-miR-549a expression in 786-O/786-O-SR cells. **(D)** Cytosolic and nuclear XPO5 expression in 786-O and Erk2 overexpressing 786-O by Western blotting. **(E,F)** RT-PCR and gel electrophoresis of PCR products analysis of pre-miR-549a expression in 786-O/786-O-SR exosomes **(E)** and HUVECs treated with exosomes **(F)**. ^∗^*P* < 0.05, ^∗∗^*P* < 0.01, ^∗∗∗^*P* < 0.001 according to two-tailed Student’s *t*-test.

To verify whether the differential expression of miR-549a between TKI-sensitive and resistant strains of ccRCC is also regulated by the above pathways, we examined the nuclear and cytoplasmic proteins of 786-O and 786-O-SR. Compared with 786-O, the nuclear XPO5 expression of 786-O-SR was higher, while the cytoplasmic expression was less (Figure 7A). Moreover, Erk2 (44 kd) expression of 786-O-SR was significantly higher than that of 786-O, while Erk1 (42 kd) was not (Figure 7B). Subsequently, we detected pre-miR-549a levels of 786-O and 786-O-SR. Pre-miR-549a levels of 786-O-SR were significantly lower than 786-O (Figure 7C). Erk2 (Figure 7B) was overexpressed in 786-O, the nuclear expression of XPO5 increased, and the cytoplasmic expression decreased (Figure 7D). This indicates that Erk2 affect the transport of pre-miR-549a via XPO5 in ccRCC.

In addition, exosomal pre-miR-549a levels of 786-O and 786-O-SR showed a consistent trend with donor cells (Figure 7E). The pre-miR-549a levels of HUVECs after exosome treatment also changed differentially (Figure 7F). This suggests that not only mature miR-549a, but also pre-miRNAs are transported to recipient cells via exosomes. In fact, XPO5, Dicer and Argonaute-2 are all expressed in exosomes (Clancy et al., 2019), which means that pre-miR-549a can be processed and matured not only in the cytoplasm of donor cells, but also in exosomes and even in recipient cells, resulting in increased levels of miR-549a-3p/miR-549a-3p in recipient cells.

### miR-549a Achieved Positive Feedback Regulation of VEGF-VEGFR2-Erk2 Pathway in Tumor Cells via HIF1α

**FIGURE 8:**
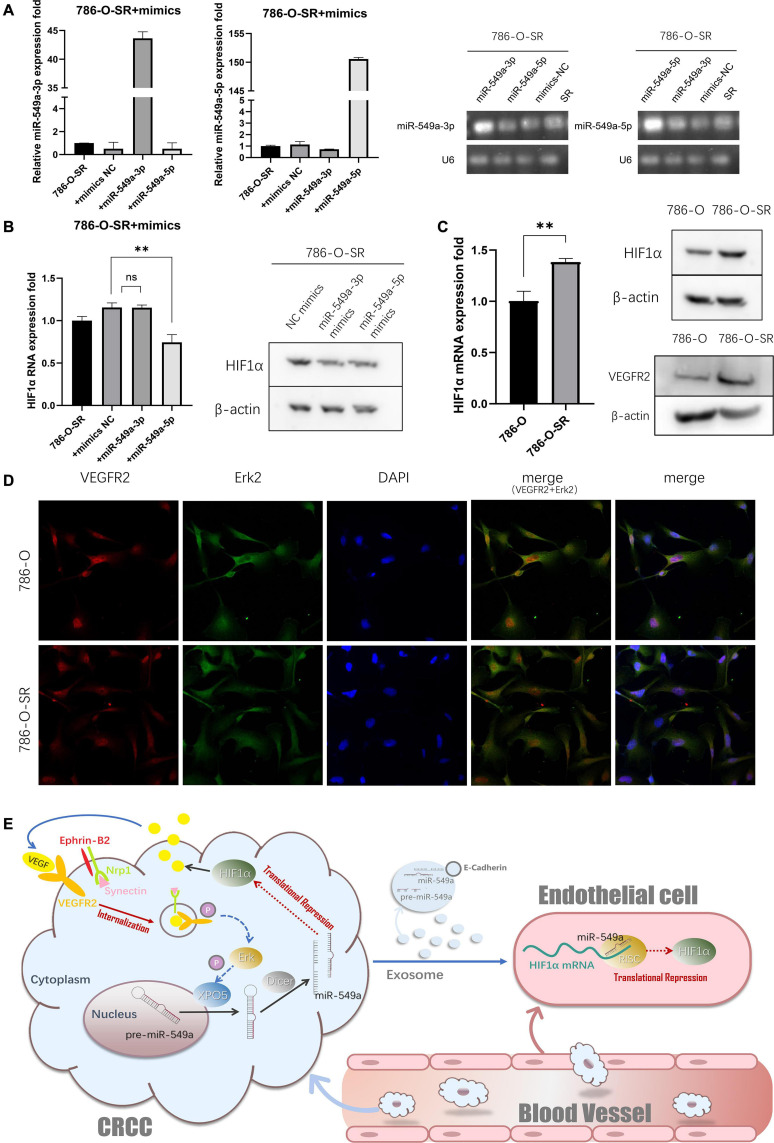
miR-549a achieved positive feedback regulation of VEGF-VEGFR2-Erk2 pathway in tumor cells via HIF1α. **(A)** RT-PCR and western blotting analysis of HIF1α expression in 786-O and 786-O-SR. VEGFR2 expression in 786-O and 786-O-SR by western blot. ^∗∗^*P* < 0.01; ns, not significant according to two-tailed Student’s *t*-test. **(B)** RT-PCR and western blotting analysis of HIF1α expression in 786-O-SR, 786-O-SR + mimics NC, 786-O-SR + miR-549a-3p mimics and 786-O-SR + miR-549a-5p mimics. **(C)** The detection of transfection efficiency of miR-549a-3p/miR-549a-5p mimics in 786-O-SR by RT-PCR and gel electrophoresis of PCR products analysis. **(D)** The subcellular localization of VEGFR2 and Erk2 was examined by confocal microscopy analysis. **(E)** Schematic diagram of the role of exosomal miR-549a in tumor metastasis and TKI resistance.

To evaluate whether the miR-549a regulatory mechanism is involved in HIF1α gene expression of ccRCC, we transfected 786-O-SR (Figure 8A) with miR549a-3p/5p mimics. After miR-549a-5p mimics transfection, both HIF1α mRNA and protein level decreased, while miR-549a-3p only had an inhibitory effect on HIF1α protein (Figure 8B), which was consistent with the experimental results of HUVEC. This indicates that there is conservation of this regulatory mechanism across different cells. Moreover, the difference of HIF1α expression between 786-O and 786-O-SR persisted after changing the cell culture medium species (Supplementary Figure 1J).

HIF1α has been demonstrated to promote the expression and secretion of VEGF (Chen et al., 2015), and the higher expression of HIF1α in 786-O-SR leads to its secretion of higher levels of VEGF than 786-O. After VEGF binding to VEGFR2 on the cell membrane of ccRCC, the multiprotein complexes, including neuropilin-1, synectin and Ephrin-B2, is initiated (Gutiérrez-González et al., 2019). Subsequently, the internalization process is initiated, and the complex is encapsulated into the membrane by intracellular endocytosis (Tian et al., 2018). After stimulation by VEGF, the subcellular localization of VEGFR2 of 786-O and 786-O-SR was located in the cytoplasm (Figure 8D), and the expression of VEGFR2 in the cytoplasm of 786-O-SR was higher than that of 786-O (Figure 8C), which confirmed that 786-O-SR had stronger VEGFR2 complex endocytosis with higher levels of VEGF. After autophosphorylation of VEGFR2, the downstream signaling pathway is activated (Clegg and Mac Gabhann, 2015). Erk, as a downstream signaling molecule of the classical VEGF pathway, is activated. A coincidence (Figure 8D) between VEGFR2 and Erk2 subcellular localization was indicated. Activated Erk2, which in turn phosphorylates XPO5, results in a decrease in the output of miR-549a. The pathway centered on miR-549a not only changes vascular permeability by exosomes acting on endothelial cells, but also affects the tumor microenvironment leading to further activation of the tumor cell VEGF pathway, forming a positive feedback regulation (Figure 8E).

## Discussion

This study revealed that exosomal miR-549a regulated the expression of HIF1α of vascular endothelial cells to promote angiogenesis, enhance vascular permeability, then promote tumorigenesis and metastasis after ccRCC TKI resistance. The regulatory effect of miR-549a on HIF1α also exists in ccRCC, which affects the secretion of VEGF, then increases the nuclear output of pre-miR-549a through the VEGFR2-ERK-XPO5 axis, forms a positive feedback. Overall, our study revealed a novel function of exosomal miR-549a and its clinical significance in TKI-resistant renal cancer.

Studies have shown that miRNAs effectively silence stromal cell mRNA via tumor cell exosomes and affect their functions (Chen et al., 2012; Zhou et al., 2014). For example, exosomal miR-103 secreted by hepatoma cells targets connexins to increase vascular permeability and promote metastasis (Fang et al., 2018). Colon cancer exosome miR-25-3p targets KLF2 and KLF4 of vascular endothelial cell, affecting their function (Zeng et al., 2018). At present, there are still few studies on miR-549a, and this study is the first to report the role of miR-549a in tumor progression. Tumor metastasis is closely related to the increase of tumor vascular permeability (Reymond et al., 2013). On the one hand, the increase of tumor vascular permeability *in situ* is conducive to the penetration of tumor cells into blood (Harney et al., 2015). On the other hand, the enhancement of vascular permeability and angiogenesis of secondary metastatic foci in distal organs provide the ‘soil’ for tumor cell colonization (Liu and Cao, 2016; Peinado et al., 2017).

As mentioned above, although the influence of tumor exosomes on vascular permeability has been proven. Whether metastasis-prone renal cancer after TKI resistance is associated with this effect remains unclear. This study revealed the interaction mechanism between tumor cells and endothelial cells mediated by exosomes. Our results showed that a series of permeability-related proteins in vascular endothelial cells were altered after co-incubation with the culture supernatants or exosomes of TKI-resistant renal cancer strains, and the degree of permeability enhancement was greater than that of sensitive strains. Therefore, we hypothesized that renal cancer cells affected vascular endothelial permeability via exosomes. After co-incubation of membrane dye-labeled exosomes with HUVEC, fluorescent signals were observed on HUVEC membranes, demonstrating the absorption of renal cancer-derived exosomes by HUVEC. Moreover, after exosome treatment, the ability of HUVEC to allow tumor cells to penetrate was enhanced. *In vivo* analyses demonstrated that renal cancer exosomes promoted tumor metastasis, and CD34-positive cell rate in tumor foci significantly increased. Array gene expression analysis of the VEGF pathway of HUVECs treated with renal cancer exosomes revealed that the change of HIF1α was significant. HIF1 is a transcription factor with helix-loop-helix structure, which is a heterodimer composed of HIF1α and HIF1β. HIF1α strongly activate the transcription and secretion of VEGF to achieve the promotion of vascular permeability and angiogenesis (Damert et al., 1997; Semenza, 2001). Our results showed that miR-549a bound to the 3′-UTR region of HIF1α mRNA to inhibit its translation process. TKI-resistant renal cancer had a weaker inhibitory effect on HIF1α due to its low expression level of miR-549a, resulting in greater vascular permeability and angiogenesis of endothelial cells. In addition, renal cancer exosomes delivered *E*-cadherin to endothelial cells to promote vascularization, but the mechanism required further studied. At present, there are many reports on the mechanism of exosomes acting on recipient cells, but there are still few studies on donor cells. This research studied the effect of miR-549a on RCC, and penetrated the signaling pathway from donor cells to receptor cells. Further studies revealed that the effect of miR-549a on HIF1α was also present in RCC cells, which in turn affected the secretion of VEGF in RCC cells. In renal cancer resistant strains, low-expression of miR-549a leads to increased secretion of VEGF by HIF1α, which induce its internalization after binding of VEGF to membrane protein VEGFR2 (Gutiérrez-González et al., 2019). Inbound VEGFR2 activates ERK, which phosphorylates XPO5 resulting in reduced pre-miR-549a output, forming a positive feedback regulation.

The enrichment in sphingolipids, phosphatidylserine, and cholesterol of exosomes has protective effect on their cargo (Skotland et al., 2019). The nucleic acid signal encapsulated by the exosome is also not easily cleared by plasma (Kamerkar et al., 2017). Changes in nucleic acid, proteins, metabolites and lipids of cancer cells are reflected in their secreted exosomes (LeBleu and Kalluri, 2020), and can be used as biomarkers for cancer early diagnosis (Jalalian et al., 2019), process monitoring (Maisano et al., 2020), and drug-resistance prediction (Liu et al., 2019; Augimeri et al., 2020). Therefore, exosomal miR-549a, which plays an important role in TKI-resistance and metastasis of RCC, may also be of value as a biomarker. Exosomes have great potential as drug delivery nano mediators due to their natural properties derived from cells (Wu et al., 2008; Schulz and Binder, 2015). Studies have shown that MSC-derived exosomes transfer miR-133b to nerve cells and can be used to treat neurodegenerative diseases (Xin et al., 2012; Yang et al., 2017). Exosomal miR-9 derived from MSC is transferred to glioblastoma multiforme and reverse its chemoresistance (Munoz et al., 2013). Overexpressed miR-let7c in MSCs is delivered into damaged renal cells, reducing renal damage in unilateral ureteral obstruction (Wang et al., 2016). Various evidences have shown that exosomes can overcome the problem that exogenous siRNAs are easy to degrade and difficult to penetrate the cell membrane, and deliver specific functional siRNAs to target cells to regulate gene expression and achieve therapeutic value. We believe that the delivery of miR-549a to TKI-resistant renal cancer cells using exosomes as carriers can effectively reduce its impact on vascular permeability and reverse its own TKI resistance.

## Data Availability Statement

The raw data supporting the conclusions of this article will be made available by the authors, without undue reservation.

## Ethics Statement

The animal study was reviewed and approved by The Laboratory Animal Center of Xiamen University (Ethics No. XMULAC20200039).

## Author Contributions

ZX and HS contributed to conception and design of the study. ZX and CC organized the database. WT performed the statistical analysis. ZS wrote the first draft of the manuscript. JZ, YZ, ZS, and LZ wrote sections of the manuscript. CS was responsible for review. All authors contributed to manuscript revision, read, and approved the submitted version.

## Conflict of Interest

The authors declare that the research was conducted in the absence of any commercial or financial relationships that could be construed as a potential conflict of interest.

## Supplementary Material

Supplementary Figure 1 Supplemental results. **(A)** RT^2^ profiler-PCR array gene expression analysis of the VEGF pathway of HUVEC cells treated with exosomes from 786-O and 786-O-SR cells. **(B)** The Table about the over-expressed and under-expressed proteins in 786-O-SR treatment group Compared with the 786-O treatment group. **(C)** Western blot analysis of *E*-cadherin expression. **(D)** The detection of transfection efficiency of miR-549a-3p/miR-549a-5p mimics/inhibitors in HUVECs by RT-PCR and gel electrophoresis of PCR products analysis. **(E)** RT-PCR and western blotting analysis of HIF1α expression. **(F)** The sequences of all primers. **(G)** The sequences of reverse transcription primers. **(H)** The sequences of miRNA mimics and inhibitors. **(I)** Western blot analysis of HIF1α expression of exosomes from 786-O and 786-O-SR. **(J)** Western blot analysis of HIF1α expression of 786-O and 786-O-SR treated by different media. ***P* < 0.01, ****P* < 0.001 according to two-tailed Student’s *t*-test.
