# Corrigendum: TKI-Resistant Renal Cancer Secretes Low-Level Exosomal miR-549a to Induce Vascular Permeability and Angiogenesis to Promote Tumor Metastasis

**DOI:** 10.3389/fcell.2021.726535

**Published:** 2021-07-19

**Authors:** Zuodong Xuan, Chen Chen, Wenbin Tang, Shaopei Ye, Jianzhong Zheng, Yue Zhao, Zhiyuan Shi, Lei Zhang, Huimin Sun, Chen Shao

**Affiliations:** ^1^Medical College, Xiamen University, Xiamen, China; ^2^School of Public Health, Xiamen University, Xiamen, China; ^3^Department of Urology Surgery, Xiang'an Hospital, Xiamen University, Xiamen, China

**Keywords:** TKI-resistant, clear cell renal cell carcinoma, exosome, microRNA, HIF1α, vascular endothelial permeability, metastasis

In the original article, there were errors. **“E-cadherin” is mistakenly stated as “N-cadherin.” Figure 2E, Figure 2F and Figure 2G are incorrectly matched to figure legends**.

A correction has been made to ***Exosomes Derived From Clear Cell Renal Cell Carcinoma Cells Increase the Permeability of the Endothelial Cells, Paragraph: 1 and 2. Exosomal miR-549a Affects Vascular Permeability. Paragraph: 4***.

To understand the effect ccRCC exert on endothelial cells and whether sorafenib-sensitive (786-O) and TKI-resistant (786-O-SR) cells have differential effects, HUVECs were cultured with CM of 786-O or 786-O-SR. After CM treatment, HUVECs showed decreased expression of β-catenin, Vimentin, ZO-1 and Claudin and up-regulated expression of E-cadherin, and the change was more significant with treatment of CM from 786-O-SR than 786-O (Figure 2A). Vimentin is a type III intermediate filament protein which plays a role in stabilizing and enhancing endothelial matrix adhesion (Tsuruta and Jones, 2003). β-catenin inhibits VE-cadherin hydrolysis (Komarova and Malik, 2010), promotes the formation and maintenance of adherent junctions. ZO-1 and Claudin are tight junction proteins. N-cadherin inhibits vascular protective repair in epithelial cells (Jian et al., 2016). The above changes indicated that the permeability of HUVECs was enhanced after CM treatment, and the effect of 786-O-SR was more obvious.

Exosome is an important tool for intercellular communication with diameters from tens to hundreds of nanometers. We extracted and identified the exosomes of 786-O and 786-O-SR. Vesicle-like structures (Figure 2B) were observed under the electron microscopy, and the expression of CD81 and TSG101 (Figure 2C) was detected by WB. The particle size of 786-O exosomes was slightly larger than that of 786-O-SR, but all were within the diameter range of exosomes (Figure 2D). After co-incubation with exosomes, the changes of β-catenin, Vimentin, ZO-1, Claudin and N-cadherin in HUVECs were the same as those after CM treatment (Figure 2F).

Transendothelial invasion assay showed that the number of 786-O-GFP crossing monolayer HUVECs increased after exosome treatment, and the effect of 786-O-SR exosome was more significant (Figure 2G). To confirm the absorption of exosomes derived from 786-O/786-O-SR by HUVECs, HUVECs were incubated with exosomes labeled with BODIPY TR ceramide, and red fluorescence signal was transferred to HUVEC (Figure 2E), but not to control group. Thus, ccRCC exosomes have an impact on vascular endothelial cell permeability, and TKI-resistant renal cancer has a greater impact on vascular permeability. However, the permeability of HUVECs treated with CM or exosomes of renal cancer cells was enhanced compared with that of the control group (i.e., HUVEC without exogenous input of miR-549a) (Figures 2A,F,G), suggesting that tumor-derived exosomes had some factors that positively regulated vascular permeability.

HUVEC naturally expressed low level of E-cadherin, a key molecule in cell-cell adhesions (van Roy and Berx, 2008), which increased after treatment with renal cancer exosomes (Figure 2F). It was reported that E-cadherin localized on the surface of exosome membrane was transported to endothelial cells to promote angiogenesis (Tang et al., 2018). E-cadherin was expressed both in 786-O/786-O-SR cells and their exosomes, and 786-O-SR expression was higher (S1C). This suggested that renal cancer exosomes transmitted E-cadherin to endothelial cells. Studies have suggested that E-cadherin regulated HIF1α (Maroni et al., 2015; Liang et al., 2016), which may be one of the mechanisms by which renal cancer exosomes promote vascular permeability.

The authors apologize for this error and state that this does not change the scientific conclusions of the article in any way. The original article has been updated.
