# Supplementary figures and images for: TKI-Resistant Renal Cancer Secretes Low-Level Exosomal miR-549a to Induce Vascular Permeability and Angiogenesis to Promote Tumor Metastasis
Source: Front Cell Dev Biol. 2021 Jun 10;9:689947. doi: 10.3389/fcell.2021.689947 (PMC8222687; doi:10.3389/fcell.2021.689947)

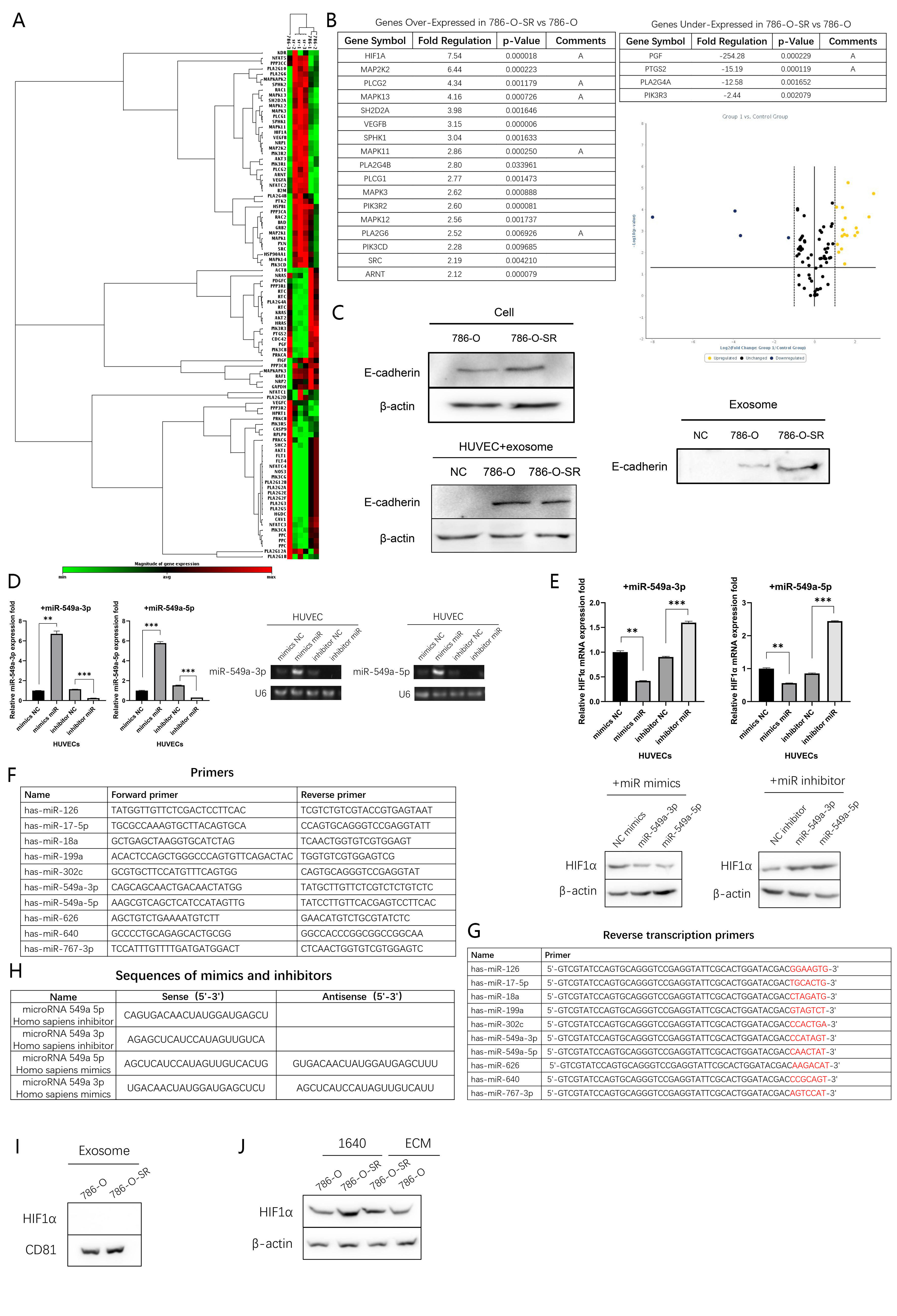

Supplement: Supplementary Figure 1 — Supplemental results. (A) RT2 profiler-PCR array gene expression analysis of the VEGF pathway of HUVEC cells treated with exosomes from 786-O and 786-O-SR cells. (B) The Table about the over-expressed and under-expressed proteins in 786-O-SR treatment group Compared with the 786-O treatment group. (C) Western blot analysis of E-cadherin expression. (D) The detection of transfection efficiency of miR-549a-3p/miR-549a-5p mimics/inhibitors in HUVECs by RT-PCR and gel electrophoresis of PCR products analysis. (E) RT-PCR and western blotting analysis of HIF1α expression. (F) The sequences of all primers. (G) The sequences of reverse transcription primers. (H) The sequences of miRNA mimics and inhibitors. (I) Western blot analysis of HIF1α expression of exosomes from 786-O and 786-O-SR. (J) Western blot analysis of HIF1α expression of 786-O and 786-O-SR treated by different media. **P < 0.01, ***P < 0.001 according to two-tailed Student’s t-test. [file Image_1.tif]
